# Super Learner Algorithm for Carotid Artery Disease Diagnosis: A Machine Learning Approach Leveraging Craniocervical CT Angiography

**DOI:** 10.3390/tomography10100120

**Published:** 2024-10-09

**Authors:** Halil İbrahim Özdemir, Kazım Gökhan Atman, Hüseyin Şirin, Abdullah Engin Çalık, Ibrahim Senturk, Metin Bilge, İsmail Oran, Duygu Bilge, Celal Çınar

**Affiliations:** 1 Department of Radiology, Faculty of Medicine, Ege University, İzmir 35100, Türkiye; halil.ibrahim.ozdemir@ege.edu.tr (H.İ.Ö.); ismail.oran@ege.edu.tr (İ.O.); celal.cinar@ege.edu.tr (C.Ç.); 2 School of Mathematical Sciences, Queen Mary University of London, London E1 4NS, UK; k.g.atman@qmul.ac.uk; 3 Department of Physics, Faculty of Science, Ege University, İzmir 35100, Türkiye; huseyin.sirin@ege.edu.tr (H.Ş.); engin.calik@ege.edu.tr (A.E.Ç.); duygu.bilge@ege.edu.tr (D.B.); 4 Department of Mathematics, Faculty of Science, Ege University, İzmir 35100, Türkiye; ibrahim.senturk@ege.edu.tr; 5 Izmir Biomedicine and Genome Center, İzmir 35340, Türkiye

**Keywords:** machine learning, carotid artery diseases, feature selection, computed tomography angiography

## Abstract

This study introduces a machine learning (ML) approach to diagnosing carotid artery diseases, including stenosis, aneurysm, and dissection, by leveraging craniocervical computed tomography angiography (CTA) data. A meticulously curated, balanced dataset of 122 patient cases was used, ensuring reproducibility and data quality, and this is publicly accessible at (insert dataset location). The proposed method integrates a super learner model which combines adaptive boosting, gradient boosting, and random forests algorithms, achieving an accuracy of 90%. To enhance model robustness and generalization, techniques such as k-fold cross-validation, bootstrapping, data augmentation, and the synthetic minority oversampling technique (SMOTE) were applied, expanding the dataset to 1000 instances and significantly improving performance for minority classes like aneurysm and dissection. The results highlight the pivotal role of blood vessel structural analysis in diagnosing carotid artery diseases and demonstrate the superior performance of the super learner model in comparison with state-of-the-art (SOTA) methods in terms of both accuracy and robustness. This manuscript outlines the methodology, compares the results with state-of-the-art approaches, and provides insights for future research directions in applying machine learning to medical diagnostics.

## 1. Introduction

Strokes, a major global health issue, rank as the second leading cause of death and a top cause of disability worldwide [1]. They occur when the blood supply to the brain is interrupted either by a blockage in a blood vessel (ischemic stroke) or a rupture (hemorrhagic stroke), leading to damage in brain cells and severe outcomes such as paralysis, speech difficulties, and cognitive impairment [2]. The likelihood of a stroke is influenced by several risk factors, including high blood pressure, smoking, obesity, diabetes, and high cholesterol, which are modifiable through lifestyle changes and medical interventions. Other risk factors, such as age, gender, and family history, are non-modifiable and require careful monitoring [3,4]. Prevention, early recognition of symptoms, and prompt medical intervention are essential to mitigating a stroke’s severe impact [5].

Carotid artery diseases, most notably stenosis, aneurysms, and dissections, are primary contributors to strokes. Stenosis, characterized by narrowing of the arteries due to plaque buildup, is a leading cause of ischemic strokes, especially when the blockage exceeds 70% [6]. Aneurysms, which involve bulging of the artery wall, pose a risk of hemorrhagic stroke if they rupture. Dissections, where tears in the artery walls lead to blood-filled channels, also increase the risk of stroke [7]. The timely diagnosis and treatment of these conditions are vital to reducing stroke incidence and preventing complications.

The structural morphology of the carotid arteries plays a significant role in the development of these diseases. The carotid artery consists of three layers: the intima, media, and adventitia. Damage to the innermost layer, the intima, can lead to the formation of atherosclerotic plaques, narrowing the artery and impeding blood flow. The common carotid artery (CCA) branches into the internal carotid artery (ICA) and external carotid artery (ECA), and their branching patterns influence the development of arterial plaques [8,9]. Understanding these structural dynamics is crucial in diagnosing and predicting carotid artery diseases.

Several well-established laws help quantify and analyze the characteristics of blood vessels and their flow dynamics. Murray’s laws, for example, describe how blood vessels optimize their structure to minimize the energy required for blood flow [8,9]. Hook’s laws further describe the relationship between the flow rate and vessel diameter, providing insights into vascular resistance and blood pressure. These principles, along with the minimum cost function, help researchers understand the structural and functional characteristics of blood vessels [10,11,12,13].

Given the complexity of carotid artery diseases, detailed analysis of blood flow dynamics is essential. Properties like branch angles, artery diameters, blood viscosity, and flow velocity are critical factors in the onset of a stroke. Cranio-cervical computed tomography angiography (CTA) allows for precise measurement of these properties, aiding in the diagnosis of arterial abnormalities such as stenosis and aneurysms [14]. Studies have shown that geometrical features, such as the angle of the ICA, can increase the risk of stroke, particularly when the angle exceeds 25 degrees [15,16]. Similarly, differences in artery diameter, influenced by factors like sex and age, have been correlated with stroke risk [17]. Ozdemir et al. identified that patients with aneurysms show larger bifurcation angles and artery diameters than healthy individuals, highlighting the role of structural deformation in disease progression [18]. Conversely, stenosis and dissection appear to have little effect on the arterial structure, although dissection is triggered by the artery diameter [19].

Artificial intelligence (AI), particularly machine learning (ML), has become increasingly influential in the diagnosis of complex diseases, including carotid artery disease (CAD). ML systems excel at identifying patterns from vast datasets without the need for explicit programming, significantly enhancing diagnostic accuracy in areas where traditional observational methods may fall short. In the context of carotid artery diseases, ML and deep learning (DL) techniques applied to carotid CT angiography (CTA) have shown remarkable potential. For instance, radiomics-based analysis of CTA images has demonstrated a superior ability to distinguish symptomatic from asymptomatic patients, achieving area under the curve (AUC) scores as high as 0.96 and outperforming conventional calcium scoring methods [20,21,22].

The success of ML models in CAD diagnosis relies heavily on well-structured and relevant feature sets, which are crucial for both the accuracy and interpretability of these models [23,24]. By extracting detailed patterns from CTA images, ML systems provide insights beyond what human experts can readily observe, aiding in better stroke risk stratification and the identification of high-risk plaques. Consequently, the integration of ML with CTA offers a promising avenue for advancing CAD diagnostics, especially in craniocervical assessments [23].

In [20], a study analyzing 132 carotid arteries from both symptomatic (ischemic stroke and transient ischemic attack) and asymptomatic patients utilized carotid CT angiography (CTA) images to detect carotid artery disease (CAD). The study revealed a small but meaningful difference in the performance of advanced imaging techniques compared with traditional methods. Radiomics achieved a mean AUC of 0.96 for distinguishing symptomatic from asymptomatic arteries, while deep learning (DL) followed closely with a mean AUC of 0.86. The calcium score, determined using the Agatston method, obtained a mean AUC of 0.79. Although the performance gap between radiomics and DL was relatively minor at ±0.02, radiomics demonstrated a slightly higher accuracy. Furthermore, for multi-class classification, radiomics showed a stronger performance with a mean AUC of 0.95, compared with 0.79 for DL. With 132 carotid arteries assessed (41 culprit, 41 non-culprit, and 50 asymptomatic), the results suggest that while DL techniques hold promise, radiomics offers a slight advantage in precision. This highlights the importance of examining even small methodological variations, as they can lead to significant improvements in diagnostic accuracy.

The main aim of this study is to develop a super learner model which integrates Adaptive Boosting, gradient boosting, and random forests algorithms to enhance the diagnostic accuracy for carotid artery diseases. By identifying and selecting relevant features from cranio-cervical CTA images, our approach offers an effective tool for diagnosing these diseases and improving health outcomes. Moreover, we provide a publicly accessible dataset to facilitate further research and reproducibility within the field. The proposed model demonstrates superior accuracy compared with existing methods, underscoring the potential of ML in medical diagnostics.

This manuscript is organized as follows. Section 2 outlines the materials and methods, including the data collection process and the machine learning algorithms utilized. In Section 3, we present techniques such as k-fold cross-validation, bootstrapping, data augmentation, and the SMOTE to enhance model robustness and generalization, particularly for datasets with limited instances. The super learner ensemble model, which integrates XGBoost, random forests, and AdaBoost, achieves a final accuracy of 90%, demonstrating superior performance. Section 4 details the results, including the performance metrics of the super learner model and its comparison with state-of-the-art approaches. Section 5 addresses the application of the SMOTE to balance the dataset by generating 880 additional synthetic samples, increasing the dataset size from 120 to 1000 instances. The SMOTE-augmented dataset retained medical plausibility, improved model generalization, and significantly enhanced performance for minority classes, such as aneurysm and dissection cases. In Section 6, we compare the model accuracy between the original and SMOTE-expanded datasets. Section 7 further compares the performance between the original dataset and the SMOTE-augmented dataset, which was optimized using Optuna. Finally, Section 8 discusses the findings in relation to the existing literature and highlights potential future research directions which could open new perspectives in the development of robust logical frameworks in the context of machine learning applications.

## 2. Materials and Methods

In this section, we employ several statistical and machine learning techniques to analyze and interpret our data effectively. The Pearson correlation coefficient (PCC) was used to measure the linear correlation between two variables, providing a quantifiable indication of the strength and direction of their relationship. This was crucial for identifying the highly correlated features in our dataset. The chi-squared test was implemented to assess the association between categorical variables, enabling us to determine if the observed frequencies deviated significantly from the expected frequencies under the null hypothesis of independence.

To ensure our data were on a comparable scale, we applied standard deviation normalization, also known as z-score normalization. This technique transformed the data to have a mean of zero and a standard deviation of one, thereby standardizing the contribution of each feature to the analysis. Lasso regularization was employed for feature selection and regularization, adding a penalty to the absolute size of the regression coefficients to improve prediction accuracy and model interpretability.

Recursive feature elimination (RFE) was utilized as a robust feature selection method, iteratively fitting a model and removing the least important features to enhance the model’s performance. This method, along with embedded methods like random forests and gradient-boosted trees, helped in identifying the most significant predictors by ranking features based on their importance.

Various machine learning algorithms, including Extreme Gradient Boosting (XGBoost), Light Gradient-Boosting Machine (LightGBM), random forests (RF), bootstrap aggregation (Bagging), Adaptive Boosting (AdaBoost), and Extremely Randomized Trees (ExtraTrees), were employed to build and optimize the model for detecting carotid artery diseases. Each algorithm was evaluated using performance metrics such as the accuracy, precision, recall, and F score. The super learner model, an ensemble of these optimized algorithms, was developed to improve the predictive accuracy by leveraging the strengths of individual models. Data collection involved obtaining CTA images from 122 patients, which were then analyzed to measure the physical properties of the carotid arteries. Feature selection mechanisms combined multiple methods to identify relevant predictors, and standard deviation normalization ensured consistent scaling of the data.

### 2.1. Pearson Correlation Coefficient

The Pearson correlation coefficient (PCC) is a measure of the linear correlation between two variables *X* and *Y* [25]. It quantifies the degree to which a linear relationship exists between them. The PCC is calculated using the formula

(1) $$r_{xy}=\frac{\sum_{i=1}^{n}\left(X_{i}-\bar{X}\right)\left(Y_{i}-\bar{Y}\right)}{\sqrt{\sum_{i=1}^{n}{\left(X_{i}-\bar{X}\right)}^{2}}\sqrt{\sum_{i=1}^{n}{\left(Y_{i}-\bar{Y}\right)}^{2}}}.$$

In this equation, $X_{i}$ and $Y_{i}$ are the individual data points, while $\bar{X}$ and $\bar{Y}$ represent the mean values of *X* and *Y*, respectively. The PCC value ranges between −1 and 1, where values closer to 1 indicate a strong positive correlation, values closer to −1 indicate a strong negative correlation, and values near 0 suggest no linear correlation.

### 2.2. Chi-Squared Test

The chi-squared test is a statistical method used to assess whether there is a significant association between two categorical variables [26]. The test statistic is calculated as follows:

(2) $$\chi^{2}=\sum_{i=1}^{n}\frac{{\left(O_{i}-E_{i}\right)}^{2}}{E_{i}}.$$

Here, $O_{i}$ represents the observed frequency, and $E_{i}$ represents the expected frequency under the null hypothesis of independence. This test is particularly useful in categorical data analysis, where the goal is to determine if the distribution of the sample categorical data matches an expected distribution.

### 2.3. Standard Deviation Normalization (Z-Score)

Standard deviation normalization, or z-score normalization, is a method used to normalize data by transforming the features to have a mean of 0 and a standard deviation of 1 [27]. The z-score for a given data point *x* is computed as follows:

(3) $$Z=\frac{x-\mu }{\sigma }$$

In this formula, *x* is the value to be normalized, μ is the mean of the dataset, and σ is the standard deviation of the dataset. This transformation ensures that each feature contributes equally to the analysis.

### 2.4. Feature Importance via Lasso Regularization

Lasso regularization is a type of linear regression which includes a penalty term for the absolute size of the regression coefficients [28]. It performs both variable selection and regularization to enhance the prediction accuracy and interpretability of the statistical model it produces. The lasso objective function is

(4) $$\min_{\beta }\left\{\frac{1}{2n}\sum_{i=1}^{n}{\left(y_{i}-\beta_{0}-\sum_{j=1}^{p}\beta_{j}x_{ij}\right)}^{2}+\lambda \sum_{j=1}^{p}\left|\beta_{j}\right|\right\}$$

Here, $y_{i}$ is the dependent variable, $x_{ij}$ represents the independent variables, $\beta_{j}$ represents the model coefficients, *n* is the number of observations, *p* is the number of predictors, and λ is the regularization parameter.

### 2.5. Recursive Feature Elimination (RFE)

Recursive feature elimination (RFE) is a feature selection method which fits a model and removes the weakest feature (or features) recursively until the specified number of features is reached [29]. The algorithm proceeds as follows:

1. Fit the model to the data.
2. Rank the features based on their importance.
3. Remove the least important feature(s).
4. Repeat the process until the desired number of features is obtained.

### 2.6. Machine Learning Models

Several machine learning algorithms were used to build and optimize the model for detecting carotid artery diseases. These algorithms include the following:

- **Extreme Gradient Boosting (XGBoost):** This algorithm uses gradient boosting framework which improves the performa nce of decision trees by combining multiple weak models.
- **Light Gradient Boosting Machine (LightGBM):** This is an efficient and effective gradient boosting framework which uses decision tree algorithms. It is designed for quick and accurate model training.
- **Random forests (RF):** This is an ensemble learning method which operates by constructing multiple decision trees during training and outputting the mode of the classes.
- **Bootstrap aggregation (bagging):** This method reduces variance by training multiple models on different subsets of the data and averaging their predictions.
- **Adaptive Boosting (AdaBoost):** This is a boosting technique which combines multiple weak classifiers to create a strong classifier.
- **Extremely Randomized Trees (ExtraTrees):** This method randomizes the choice of the split point and features to reduce variance in high-dimensional data.

The performance of each model was evaluated using metrics such as the accuracy, precision, recall, and F score. The models were optimized using Optuna optimization software to find the best hyperparameters for each algorithm.

A super learner model was developed by combining the optimized versions of the individual machine learning algorithms. This ensemble model was designed to improve the predictive accuracy by leveraging the strengths of each algorithm. The final super learner model was constructed by combining the XGBoost, random gorest, and bagging algorithms, achieving an overall accuracy of 0.90.

### 2.7. Data Collection

**Figure 1:**
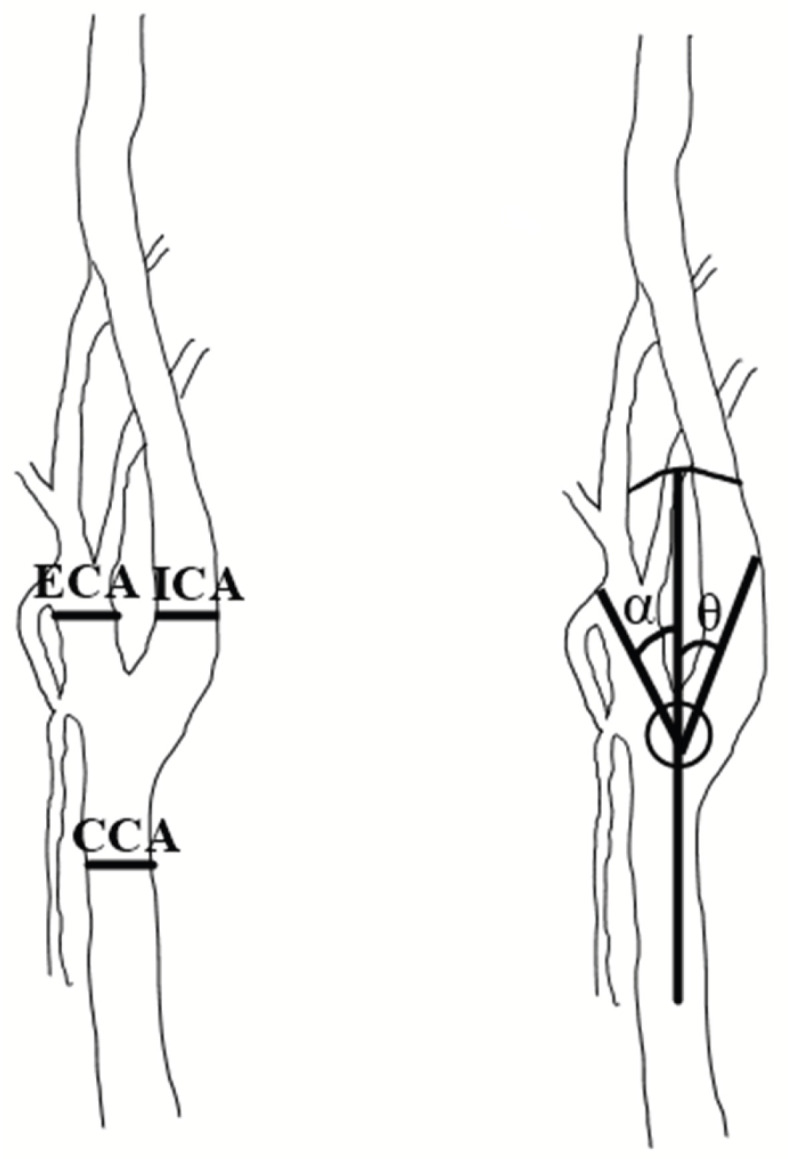
Illustration of the CCA, ICA, and ECA values and bifurcation angles of the vessels.

**Figure 2:**
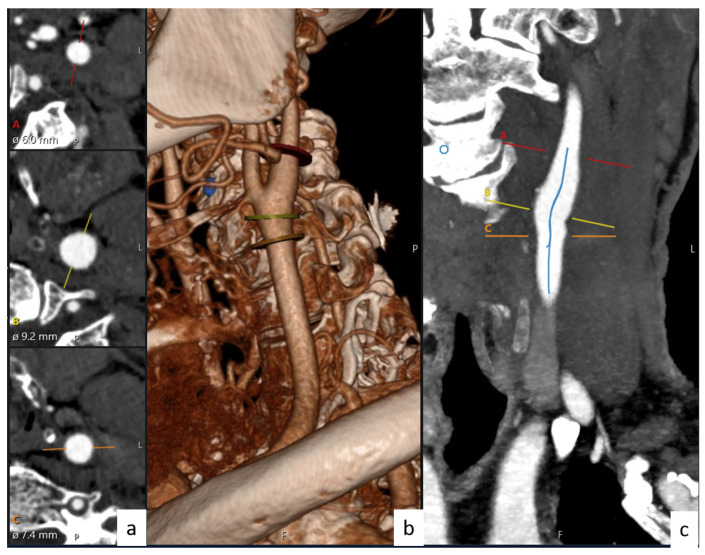
Diameters and angles of carotid artery with the help of Sectra software. (**a**) Diameter, (**b**) 3D imaging of carotid arteries, and (**c**) artery lumen centers.

**Figure 3:**
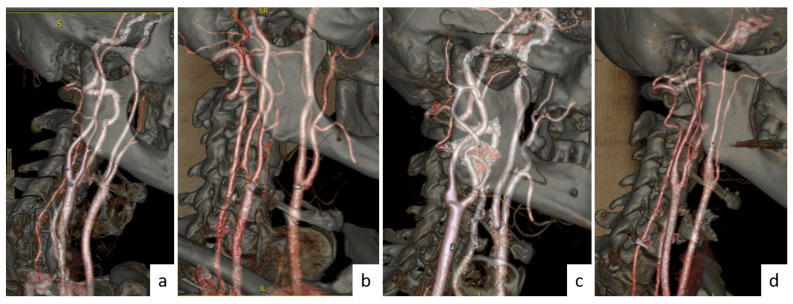
Set of 3D images of the carotid arteries obtained with AW Server software for patients: (**a**) normal, (**b**) stenosis, (**c**) aneurysm, and (**d**) dissection.

In this study, CTA images from 122 patients obtained from Ege University Hospital (protocol number/code: 19-9.1T/6) were selected to measure some physical properties of the vessels. These CTA images belong to diseases classified as stenosis (30 persons), aneurysm (30 persons), and dissection (31 persons). As an addition, the persons without disease (31 persons) were evaluated as normal. In accordance with these data, CTA was used to obtained the diameters and angles of the CCA, ICA, and ECA. Here, CCA, ICA, and ECA are abbreviations of the diameters of the common, internal, and external carotid arteries, respectively. The illustrations of these CCA, ICA, and ECA branches and bifurcation angles between them are shown in Figure 1. The CCA, ICA, and ECA and bifurcation angle values of all persons (normal and patients) were measured with the help of CTA images with a great accuracy. The angles and the diameter values for all patients (normal, stenosis, aneurysm, and dissection) were obtained by taking the bisectors of the ICA, ECA, and CCA and from the bulber segments, respectively. The values obtained from every patient were taken more than once, and their averages were used for the calculations. As shown in Figure 2 and Figure 3, the images obtained from CTA were analyzed with the help of Sectra (Sectra Workstation IDS7 for Windows Version, Sectra AB, Sweden) and AW Server 2 (AW SERVER 2.0 EXT. 7.1 SOFTWARE AND DOCS DVD by GE Healthcare, Chicago, IL, USA) programs. The parameters used in CTA were kVp, mA, rotation time, section thickness, pitch value, coverage, kernel filter, medium, matrix, and FOV, and their values were 120, dose modulated, 0.3 s, 0.6 mm, 0.8, 76.8 mm, 326 f, smooth, 512×512, and 230 mm, respectively [18,30].

### 2.8. Feature Extraction and Preprocessing

To identify the most significant features from the dataset, we employed a comprehensive feature selection (FS) mechanism which integrates three types of feature selection algorithms: filter-based, embedded, and hybrid. Each approach has its advantages and drawbacks, and by combining them, we aimed to enhance the accuracy and reduce the complexity of the machine learning (ML) model [31]:

- **Filter-based FS algorithms** are statistical methods which rank features independent of their relationships with the target variable [32]. One such method is the correlation filter, which uses the Pearson correlation coefficient (PCC) to identify highly correlated features. The PCC is calculated with the help of Equation (1). Here, $X_{i}$ and $Y_{i}$ are individual data points, while $\bar{X}$ and $\bar{Y}$ are their respective mean values. PCC values closer to 1 indicate a strong positive relationship, values closer to −1 indicate a strong negative relationship, and values near 0 suggest no linear correlation [33]. Additionally, the chi-squared test was used to measure the dependence among features, which is particularly useful for categorical data.
- **Embedded methods** incorporate feature selection as part of the model training process. These methods use a wrapper to evaluate the importance of features without building a new model each time a different subset is selected. In our study, we used lasso regularization, random forests, and gradient-boosted trees to determine feature importance. Lasso regularization, for instance, minimizes the following objective function obtained with the help of Equation (4). This method penalizes the absolute size of the coefficients, effectively shrinking some of them to zero and thus performing variable selection and regularization simultaneously.
- **Hybrid methods** combine the strengths of both filter-based and embedded methods. Recursive feature elimination (RFE) is a hybrid method we used which iteratively fits a model and removes the least important feature(s) until the specified number of features is reached. The process involves the following:
  1. Fitting the model to the data.
  2. Ranking the features based on their importance.
  3. Removing the least important feature(s).
  4. Repeating the process until the desired number of features is obtained.

RFE is a greedy optimization algorithm which creates models repeatedly to find the best performing subset by excluding the least important feature at each iteration.

To further refine the model, Table 1, Table 2, Table 3 and Table 4 provide detailed demographic and clinical measurements of four different groups of cases: normal, stenosis, aneurysm, and dissection, respectively. The demographic characteristics in each table include key attributes such as the age, measured angle data, and diameters of various arterial regions. These tables include both male and female participants, with a variety of minimum, maximum, mean, and standard deviation (STD) values for each attribute.

- **Normal cases**: The demographic structure of the study’s normal sample included a total of 31 participants, with 16 females and 15 males. The ages of the participants ranged from 30 to 79 years, with a mean age of 55.45 years and a standard deviation of 14.16, indicating a moderately diverse age range. The difference between the measured $\cos\theta^{\prime }$ and the predicted cosθ values (based on Murray’s law) varied between −0.456 and 0.165, with a mean of −0.102 and a standard deviation of 0.153, suggesting a slight tendency for the measured values to be lower than predicted.
  Regarding anatomical measurements, the right internal carotid artery diameter (ICAdiaR) varied from 4.4 mm to 8.0 mm, with a mean of 5.703 mm and a standard deviation of 0.812, reflecting relatively low variability. The left external carotid artery angle (Car.Angle.Lα) spanned from 12° to 58°, with an average of 27.2° and a standard deviation of 12.639, indicating notable variation. The total right carotid angles (Car.Angle.ΣR) ranged from 20.8° to 76.0°, with a mean of 46.65° and a standard deviation of 14.934, while the total left carotid angles (Car.Angle.ΣL) varied more significantly, ranging from 26.0° to 105.4°, with a mean of 50.97° and a standard deviation of 19.198. The right carotid angle (Car.Angle.θR) had values between 6.3° and 54.1°, with a mean of 23.05° and a standard deviation of 11.427, while the left carotid angle (Car.Angle.θL) ranged from 4.7° to 49.0°, with a mean of 23.77° and a standard deviation of 12.48. The combined right and left carotid angles (Car.Angle.(R+L)θ) spanned from 6.1° to 47.65°, with an average of 23.41° and a standard deviation of 10.836. Lastly, the right common carotid artery diameter (CCAdiaR) ranged from 5.1 mm to 10.7 mm, with a mean of 7.161 mm and a standard deviation of 1.049, showing relatively consistent measurements across the participants.
- **Stenosis cases**: The demographic structure of stenosis cases consists of 30 participants, with 10 females and 20 males. The ages of the participants ranged from 52 to 70 years, with a mean age of 63.73 years and a standard deviation of 4.948, indicating a somewhat narrow age range. The difference between the measured $\cos\theta^{\prime }$ and the predicted cosθ values (based on Murray’s law) varied between −0.458 and 0.093, with a mean of −0.177 and a standard deviation of 0.140, suggesting that the measured values tended to be lower than predicted.
  In terms of anatomical measurements, the right internal carotid artery diameter (ICAdiaR) ranged from 3.5 mm to 10.5 mm, with a mean of 5.707 mm and a standard deviation of 1.484, indicating notable variability. The left external carotid artery angle (Car.Angle.Lα) spanned from −67.3° to 45.0°, with an average of 16.77° and a standard deviation of 20.079, suggesting significant variation. The total right carotid angles (Car.Angle.ΣR) ranged from 22.6° to 66.4°, with a mean of 41.51° and a standard deviation of 10.079, while the total left carotid angles (Car.Angle.ΣL) ranged from 10.7° to 83.9°, with a mean of 42.24° and a standard deviation of 16.088, indicating more variability on the left side.
  The right carotid angle (Car.Angle.θR) ranged from 3.0° to 36.6°, with a mean of 17.44° and a standard deviation of 7.68, while the left carotid angle (Car.Angle.θL) ranged from 4.0° to 78.0°, with a mean of 25.48° and a standard deviation of 15.115, reflecting wider variability on the left side. The total right and left carotid angles (Car.Angle.(R+L)θ) ranged from 9.35° to 53.7°, with a mean of 23.46° and a standard deviation of 9.511. Lastly, the right common carotid artery diameter (CCAdiaR) spanned from 5.9 mm to 11.1 mm, with a mean of 7.748 mm and a standard deviation of 1.898, showing relatively consistent measurements.
- **Aneurysm cases**: The demographic structure of aneurysm cases consists of 30 participants, with 13 females and 17 males. The ages of the participants ranged from 33 to 74 years, with a mean age of 53.17 years and a standard deviation of 11.68, indicating moderate age variation. The difference between the measured $\cos\theta^{\prime }$ and the predicted cosθ values (based on Murray’s law) varied between −0.391 and 0.764, with a mean of 0.101 and a standard deviation of 0.258, showing greater deviation compared with the other cases.
  In terms of anatomical measurements, the right internal carotid artery diameter (ICAdiaR) ranged from 3.6 mm to 9.4 mm, with a mean of 6.693 mm and a standard deviation of 1.371, indicating a wider variation in the artery diameter. The left external carotid artery angle (Car.Angle.Lα) ranged from 0.0° to 46.9°, with an average of 21.08° and a standard deviation of 11.115, reflecting significant variability. The total right carotid angles (Car.Angle.ΣR) spanned from 29.2° to 125.0°, with a mean of 53.997° and a standard deviation of 19.547, while the total left carotid angles (Car.Angle.ΣL) ranged from 24.1° to 102.6°, with a mean of 52.317° and a standard deviation of 17.026, indicating similar variability on both sides.
  The right carotid angle (Car.Angle.θR) ranged from 4.3° to 67.1°, with a mean of 28.76° and a standard deviation of 13.267, while the left carotid angle (Car.Angle.θL) ranged from 8.1° to 63.8°, with a mean of 31.23° and a standard deviation of 14.587, reflecting higher variation. The total right and left carotid angles (Car.Angle.(R+L)θ) spanned from 8.05° to 61.4°, with a mean of 29.998° and a standard deviation of 12.894. Lastly, the right common carotid artery diameter (CCAdiaR) ranged from 4.9 mm to 10.8 mm, with a mean of 7.393 mm and a standard deviation of 1.353, showing relatively consistent measurements across the participants.
- **Dissection cases**: The demographic structure of the dissection cases included 31 participants, with 18 females and 13 males. The ages of the participants ranged from 27 to 76 years, with a mean age of 48.71 years and a standard deviation of 10.558, reflecting moderate age variation. The difference between the measured $\cos\theta^{\prime }$ and the predicted cosθ values (based on Murray’s law) ranged from −0.429 to 0.256, with a mean of −0.817 and a standard deviation of 0.177, indicating a tendency for the measured values to be lower than predicted.
  Regarding anatomical measurements, the right internal carotid artery diameter (ICAdiaR) spanned from 3.3 mm to 6.7 mm, with a mean of 4.877 mm and a standard deviation of 0.978, showing moderate variability. The left external carotid artery angle (Car.Angle.Lα) ranged from 1.0° to 61.2°, with a mean of 22.094° and a standard deviation of 12.735, reflecting significant variation. The total right carotid angles (Car.Angle.ΣR) varied between 23.8° and 99.9°, with a mean of 49.629° and a standard deviation of 16.831, while the total left carotid angles (Car.Angle.ΣL) spanned from 25.2° to 93.8°, with a mean of 51.545° and a standard deviation of 16.909, indicating similar variability on both sides.
  The right carotid angle (Car.Angle.θR) ranged from 7.2° to 40.8°, with a mean of 23.516° and a standard deviation of 8.299, while the left carotid angle (Car.Angle.θL) varied from 7.4° to 55.9°, with a mean of 29.452° and a standard deviation of 12.408, showing greater variation on the left side. The total right and left carotid angles (Car.Angle.(R+L)θ) ranged from 12.7° to 46.85°, with a mean of 26.484° and a standard deviation of 9.458. Lastly, the right common carotid artery diameter (CCAdiaR) ranged from 4.9 mm to 8.9 mm, with a mean of 6.261 mm and a standard deviation of 0.888, indicating relatively consistent measurements across the participants.

Finally, Table 5 is a summary table which combines the data from all four previous tables. This table provides an overall demographic overview of the entire dataset. The general demographic structure included a total of 122 participants, with 57 females and 65 males. The participants’ ages ranged from 27 to 79 years, with a mean age of approximately 54.84 years and a standard deviation of around 12.34, reflecting a relatively broad age distribution. The difference between the measured $\cos\theta^{\prime }$ and the predicted cosθ values varied from −0.458 to 0.764 across the cases, with an overall mean of approximately −0.248 and a standard deviation of 0.189, indicating a tendency for the measured values to be lower than the predicted values. The measured cosθ values spanned from 0.479 to 0.994, with a mean of 0.887 and a standard deviation of 0.090. The right internal carotid artery diameter (ICAdiaR) ranged from 3.3 mm to 10.5 mm, with an overall mean of 5.745 mm and a standard deviation of 1.161, indicating moderate variation.

The left external carotid artery angle (Car.Angle.Lα) varied significantly from −67.3° to 61.2°, with a mean of 21.03° and a standard deviation of 14.067. The total right carotid angles (Car.Angle.ΣR) spanned from 20.8° to 125.0°, with a mean of 47.47° and a standard deviation of 15.848, while the total left carotid angles (Car.Angle.ΣL) ranged from 10.7° to 105.4°, with a mean of 49.52° and a standard deviation of 17.305. The right carotid angle (Car.Angle.θR) ranged from 3.0° to 67.1°, with a mean of 23.74° and a standard deviation of 10.668, while the left carotid angle (Car.Angle.θL) spanned from 4.7° to 78.0°, with a mean of 27.23° and a standard deviation of 13.148. The combined right and left carotid angles (Car.Angle.(R+L)θ) ranged from 6.1° to 61.4°, with a mean of 25.34° and a standard deviation of 10.74. Lastly, the right common carotid artery diameter (CCAdiaR) ranged from 4.9 mm to 11.1 mm, with an overall mean of 6.641 mm and a standard deviation of 1.214, reflecting consistent measurements across the participants.

This general overview offers a comprehensive summary of the variability and commonalities observed across the cases.

The primary goal of our feature selection approach was to identify common significant features selected by all the employed methods. By accomplishing this, we ensured that the training dataset included only the most relevant features, thereby improving the model’s interpretability and performance. At this point, it should be also noted that the dataset was split into training and test sets prior to feature selection to ensure the integrity of the model evaluation and avoid data leakage.The relevant predictors for diagnosing carotid artery diseases are summarized in Table 5.

Since the variables for carotid artery diseases are sparse, and the presence of outliers may negatively affect the predictive performance of ML models, scaling the data is essential. We accomplished this by applying standard deviation normalization (z-score normalization), which scales each feature by dividing its values by the standard deviation. This transformation ensures that all features contribute equally to the analysis. The z-score was calculated as shown in Equation (3), where x is the data point, μ is the mean, and γ is the standard deviation. This normalization process is discussed in more detail in Section 2.3.

## 3. Enhanced Model Robustness Techniques

To mitigate the challenges posed by the relatively limited dataset, we implemented several advanced techniques to improve the model robustness and accuracy. These included k-fold cross-validation, bootstrapping, data augmentation, and synthetic data generation using the SMOTE.

### 3.1. Cross-Validation

We employed *k-fold cross-validation* to optimize the model’s validation process. Specifically, we used five-fold cross-validation, where the dataset was divided into five subsets. In each iteration, the model was trained on four subsets and validated on the remaining subset. This process was repeated five times, ensuring that each subset was used as a validation set once. The following Python code was used for the implementation:

from sklearn.model_selection import KFold
kf = KFold(n_splits=5)
for train_index, test_index in kf.split(X):
X_train, X_test = X[train_index], X[test_index]
y_train, y_test = y[train_index], y[test_index]
model.fit(X_train, y_train)
predictions = model.predict(X_test)

### 3.2. Bootstrapping

We used *bootstrapping* to create multiple resampled versions of the training data, thereby increasing the number of training examples and allowing for better model generalization. This is particularly helpful in a small dataset scenario, where variance reduction is crucial. Bootstrapping was implemented as shown below:

from sklearn.utils import resample
X_bootstrap, y_bootstrap = resample(X_train, y_train,
replace=True,
n_samples=len(X_train))
model.fit(X_bootstrap, y_bootstrap)

### 3.3. Data Augmentation

Although data augmentation is typically applied in image classification tasks, we adapted this technique to our dataset by introducing random perturbations to key features, such as artery diameters and bifurcation angles. Gaussian noise was added to simulate small variations, which increased the training data’s diversity:

import numpy as np
noise = np.random.normal(0, 0.01, X_train.shape)
X_augmented = X_train + noise
model.fit(X_augmented, y_train)

### 3.4. Synthetic Data Generation (SMOTE)

We applied the *synthetic minority oversampling technique (SMOTE)* to generate synthetic examples for the minority classes in our dataset, such as aneurysms and dissections. This technique helped preserve the balance of the dataset by creating new samples from the existing data:

from imblearn.over_sampling import SMOTE
smote = SMOTE()
X_resampled, y_resampled = smote.fit_resample(X_train, y_train)
model.fit(X_resampled, y_resampled)

### 3.5. Ensemble Learning

We enhanced the robustness of the model by utilizing an ensemble learning approach. The *super learner* model combines optimized versions of XGBoost, random forests, and AdaBoost, among other algorithms. This ensemble method leverages the strengths of each algorithm to achieve superior performance:

from sklearn.ensemble import StackingClassifier
estimators = [
(’rf’, RandomForestClassifier(n_estimators=100)),
(’xgb’, XGBClassifier()),
(’ada’, AdaBoostClassifier())
]
stacking_model = StackingClassifier(estimators=estimators,
final_estimator=LogisticRegression())
stacking_model.fit(X_train, y_train)

After implementing these robustness-enhancing techniques, the final super learner model demonstrated a significant improvement in accuracy and robustness, with a final accuracy of 90%. The integration of cross-validation, bootstrapping, data augmentation, and the SMOTE contributed to this result by ensuring better generalization across various datasets and reducing overfitting.

## 4. Construction of Carotid Artery Disease Detection Model

The difficulties encountered by systems which rely on hard-coded knowledge indicate that artificial intelligence (AI) systems require the capability to learn independently by extracting patterns from raw data [23]. This capability is referred to as machine learning (ML). ML is a type of applied statistics which uses computers to statistically estimate complex functions, enabling them to solve real-world problems and make decisions that appear subjective. The main aim of this study is to investigate the applicability of different ML techniques in detecting carotid artery diseases.

We began by analyzing various types of ML algorithms and tuning their hyperparameters using the optimization software *Optuna* (https://github.com/pfnet/optuna/ accessed on 26 September 2024) [34]. After optimizing the candidate algorithms, we built a super learner model by creating a weighted combination of these candidates. The following ML algorithms were considered candidate learners:

- **Extreme Gradient Boosting (XGBoost)**: XGBoost is a supervised ML algorithm based on the tree boosting method [35]. It is an ensemble learning algorithm which creates a final model from a collection of individual models, typically decision trees. XGBoost uses gradient descent to optimize weights and minimize the loss function, considering second-order gradients to improve model performance.
- **Light Gradient-Boosting Machine (LightGBM)**: LightGBM is a variant of gradient boosting which achieves superior performance, especially with high-dimensional data and large datasets [36]. It employs two novel techniques—Gradient-based one-side sampling (GOSS) and exclusive feature bundling (EFB)—which enhance the training speed and efficiency. Like XGBoost, LightGBM is based on decision tree algorithms.
- **Random forests (RF)**: RF is a robust ensemble learning method based on a collection of decision trees [37]. Each tree is constructed using a random vector sampled independently but with the same distribution across all trees. Nodes in the decision trees are split based on measures like entropy or the Gini index.
- **Bootstrap aggregation (bagging)**: Bagging is an ensemble learning technique which reduces variance in noisy datasets and is considered an extension of the random forests algorithm [38]. It involves selecting random samples of data with replacement, training multiple models independently, and averaging their predictions for improved accuracy.
- **Adaptive Boosting (AdaBoost)**: AdaBoost is a boosting approach which generates a robust classifier from a set of weak classifiers. It maintains weights over the training data and adjusts them adaptively after each learning cycle, increasing the weights for incorrectly classified samples and decreasing the weights for correctly classified ones.
- **Extremely Randomized Trees (ExtraTrees)**: ExtraTrees is an ensemble learning technique based on decision trees. Unlike RF, where the tree splits are deterministic, ExtraTrees uses randomized splits, providing a robust approach for high-dimensional data by balancing bias and variance.

**Figure 4:**
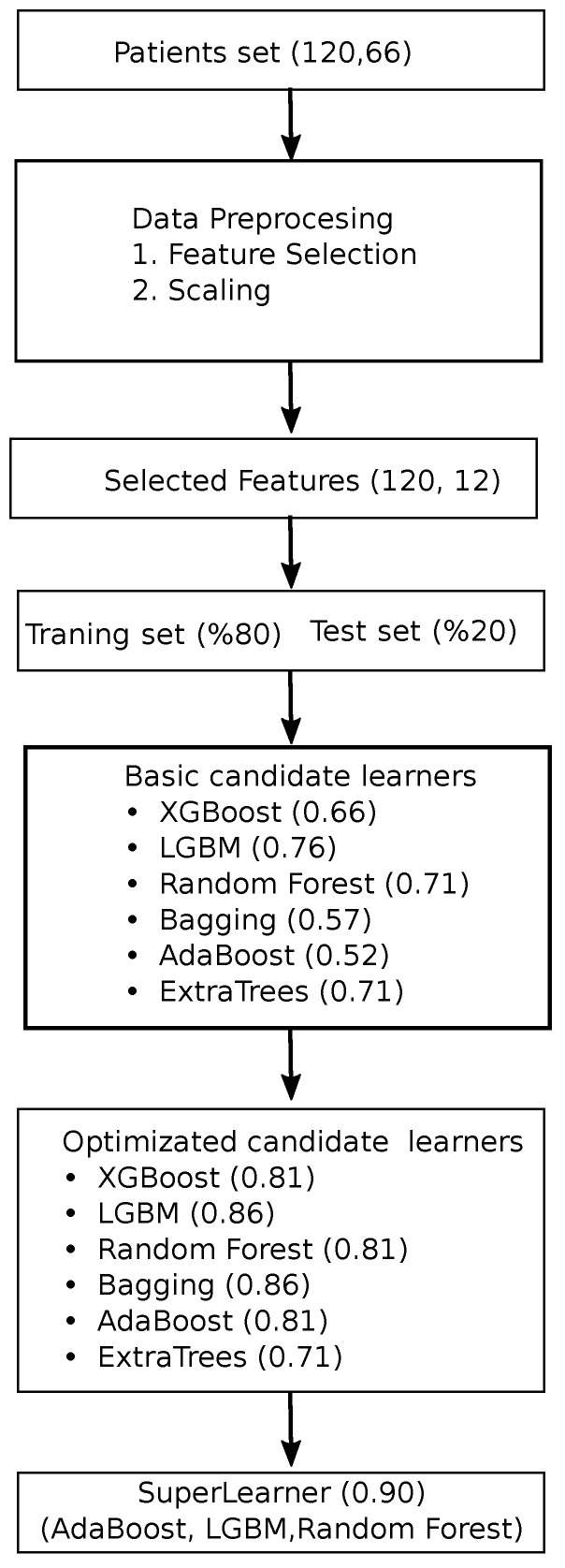
Workflow of the proposed AI-based approach.

In Figure 4, the workflow chart involves a systematic process to predict patient outcomes using machine learning techniques. Initially, a dataset comprising patient data is collected and preprocessed, where feature selection is conducted to identify the most relevant variables, reducing the dataset’s dimensionality. These selected features are then scaled to ensure uniformity across all variables. The dataset is subsequently divided into a training set and a test set, with 80% of the data used for training and 20% reserved for testing. A variety of basic machine learning models, including XGBoost, LGBM, random forests, bagging, AdaBoost, and ExtraTrees, are trained on the training set. Each model’s performance is evaluated, and optimization techniques are applied to enhance their accuracy. Following optimization, a super learner model is constructed by combining the top-performing models, specifically AdaBoost, LGBM, and random forests. The final super learner model achieved the highest performance, illustrating the value of model optimization and ensemble techniques in predictive analytics.

To establish the hyperparameter search for the ML algorithms, we employed *Optuna* optimization software, since it is an efficient hyperparameter optimization framework which tunes machine learning models by intelligently exploring the hyperparameter search space. It uses a tree-structured Parzen estimator (TPE) as a probabilistic model to guide the search, balancing exploration and exploitation. Optuna evaluates different sets of hyperparameters by running trials and selecting those which improve the model’s performance based on a specified objective, such as minimizing validation loss or maximizing accuracy. The framework also employs pruning strategies which stop unpromising trials early, reducing computational cost by focusing on the hyperparameters. The primary advantage of *Optuna* is its efficient hyperparameter search, facilitate by its *define-by-run* principle. Table 6 provides a summary of each algorithm’s performance across accuracy scores before the ensemble. According to this, AdaBoost demonstrated strength in handling weak learners but was sensitive to noisy data, which affected its overall performance. Random forests performed well in terms of avoiding overfitting and generalizing across different data subsets, although it had difficulty distinguishing fine-grained class distinctions. XGBoost was particularly effective at handling complex patterns and sparse data but required careful tuning to maximize performance. LightGBM had the highest accuracy and offered faster training times, though it was sensitive to imbalanced datasets. Bagging, with similar accuracy to LightGBM, provided reliable generalization by reducing variance but lacked the fine-tuned control of boosting algorithms. Finally, ExtraTrees, though proficient at managing noisy data due to its randomized split point selection, had reduced precision and lower overall performance. These findings highlight how combining these algorithms in an ensemble capitalizes on their complementary strengths. Bagging and random forests contributed to variance reduction and robustness, XGBoost and LightGBM exceled in managing complex datasets, AdaBoost focused on improving weak learners, and ExtraTrees managed noisy data. This ensemble approach resulted in a more generalized and accurate model than any of the individual algorithms could achieve on their own.

To achieve higher predictive accuracy, we implemented stacked generalization of the distinct candidate learners, namely in an ensemble method [39,40]. In this approach, lower-level predictive algorithms (candidate learners) are combined into a higher-level model called a super learner (SL) [41]. After developing the SL, we used *Optuna* again to find the best combination of candidate learners. The best SL model, which combined the XGBoost, RF, and bagging algorithms, achieved an overall accuracy of 0.90. Table 7 presents the performance metrics, namely the precision, recall, and F score, for the corresponding classes in the test data.

## 5. Dataset Expansion Using SMOTE

In the recent literature, it has been observed that one study [42] utilized the Nasarian Coronary Artery Disease (CAD) dataset, which incorporates both workplace and environmental factors alongside clinical features. The results demonstrated that the proposed feature selection method achieved a classification accuracy of 81.23% by employing the SMOTE technique and the XGBoost classifier. The synthetic minority oversampling technique (SMOTE) approach plays a crucial role in addressing class imbalance within the dataset, improving model performance by generating synthetic examples of the minority class and thereby ensuring a more balanced training process. This technique, which is widely used in contemporary research, significantly enhances the robustness and accuracy of machine learning models when applied to imbalanced datasets like the CAD dataset in this study.

In order to increase the amount of data for this work, we applied the synthetic minority oversampling technique (SMOTE). The original dataset consisted of 120 instances, with 80 normal cases and 40 instances of aneurysm and dissection cases, leading to scarcity. Through the SMOTE, we increased the number of minority class instances, expanding the dataset to approximately 1000 samples.

### 5.1. SMOTE Process

The SMOTE augmentation involved oversampling the minority classes by generating 880 additional synthetic samples and increasing the minority class size from 40 to 920 instances while keeping the 80 majority class samples unchanged. This resulted in a total of 1000 instances, creating and preserving a balanced dataset as in Table 8.

### 5.2. Outcome of SMOTE Application

The SMOTE application yielded the following benefits:

- Expanding the dataset to 1000 samples.
- Addressing class scarcity by increasing the minority class to 920 instances.
- Enhancing model accuracy, particularly for the minority classes.

The SMOTE-augmented dataset provided a more balanced and robust training set, improving model generalization across both the majority and minority classes.

Moreover, we employed several measures to ensure the quality and reliability of the SMOTE-augmented dataset. The generated features, such as age, carotid angles, and artery diameters, were kept within realistic ranges observed in the original data, ensuring that the augmented data maintained medical plausibility. The SMOTE generated synthetic samples by interpolating between existing minority class data points, which avoided duplication and reduced the risk of overfitting.

We validated the augmented data by comparing statistical properties such as the mean, standard deviation, minimum, and maximum values with the original dataset, ensuring consistency. Additionally, related attributes like the artery diameters and age were cross-verified to preserve the expected medical relationships. The sex and age distributions were balanced to reflect equal representation of male and female subjects, maintaining the overall demographic structure.

Finally, consistency checks were implemented throughout the augmentation process to ensure data integrity, avoiding outliers and unrealistic values. These steps resulted in a robust and reliable dataset for training and evaluating machine learning models in the diagnosis of carotid artery diseases.

## 6. Model Accuracy Comparison: Original Dataset versus SMOTE-Expanded Dataset

This section presents the performance comparison of machine learning models trained on both the original dataset and the SMOTE-expanded dataset. The evaluated models included XGBoost, random forests, bagging, AdaBoost, and ExtraTrees.

### 6.1. Model Training on Original Dataset

The original dataset consisted of 120 instances, with 80 belonging to the majority class and 40 belonging to the minority classes. The models were trained and evaluated on this original dataset, as shown in Table 9.

### 6.2. Model Training on SMOTE-Expanded Dataset

After applying the SMOTE, the minority class was increased to 920 instances, resulting in a balanced dataset of 1000 instances. The same models were trained on this expanded dataset, as shown in Table 10.

### 6.3. Comparison and Analysis

Table 11 illustrates the comparison between model accuracies before and after applying the SMOTE. The results indicate significant improvements in the models’ ability to generalize with a balanced dataset.

Application of the SMOTE resulted in marked improvements in models such as LightGBM, random forests, and bagging. For example, LightGBM’s accuracy increased from 0.76 to 0.86, and bagging saw a rise from 0.57 to 0.86. However, ExtraTrees did not exhibit any significant improvement, suggesting that this model may not benefit as much from balancing the dataset using the SMOTE.

## 7. Performance Comparison: Original versus SMOTE with Optuna

We also compared the models’ performance on both datasets after applying Optuna optimization. The SMOTE balanced the dataset, while Optuna optimized the hyperparameters of the models, leading to significant improvements.

### 7.1. Optuna Optimization on Original Data and on SMOTE-Expanded Data

Table 6 presents the accuracy scores after Optuna optimization on the original dataset.

Table 12 shows the accuracy scores for the models trained on the SMOTE-augmented dataset after Optuna optimization. Balancing the dataset with the SMOTE, coupled with efficient hyperparameter tuning via Optuna, further enhanced model performance.

### 7.2. Comparison of Performance

A performance comparison between the original dataset and the SMOTE-balanced dataset after Optuna optimization is presented in Table 13. Across all models, balancing the dataset with the SMOTE led to higher accuracies, with the most significant gains observed in models like XGBoost, LightGBM, and AdaBoost.

As a result, the comparison between the original and SMOTE-augmented datasets (Table 13) highlights how the SMOTE and Optuna together improved model accuracy. Noteworthy observations include the following:

- **XGBoost**: Accuracy increased from 0.81 to 0.89 after SMOTE and Optuna optimization.
- **LightGBM**: Accuracy improved from 0.86 to 0.91, making it the top performer.
- **Bagging and random forests**: Bagging’s accuracy increased from 0.86 to 0.90, while that of random forests improved from 0.81 to 0.88.
- **AdaBoost and ExtraTrees**: AdaBoost’s accuracy improved from 0.81 to 0.87, while that of ExtraTrees increased from 0.71 to 0.79.

Overall, the models benefited significantly from the balanced dataset provided by the SMOTE, with each model demonstrating improved performance after hyperparameter optimization with Optuna. The comparison shows that using the SMOTE, followed by Optuna optimization, enhanced model accuracy across all tested algorithms.

To further improve the predictive accuracy on the SMOTE-augmented dataset, we implemented stacked generalization of various optimized candidate learners through an ensemble technique [39,40]. This method combines lower-level models (candidate learners) into a higher-level ensemble model known as the super learner (SL) model [41]. After constructing the SL model using the SMOTE-augmented data, we employed *Optuna* once again to identify the optimal combination of candidate learners for the best performance.

The best SL model, integrating the XGBoost, random forests (RF), and bagging algorithms, achieved an overall accuracy of 0.91 on the SMOTE-augmented dataset. This model showed improved performance, especially in handling the minority classes due to the balanced nature of the data.

Table 14 summarizes the classification report, detailing the precision, recall, and F scores for the individual classes within the test data. The results highlight the improved classification performance for all classes, particularly the minority categories such as aneurysm and dissection cases, which previously suffered from scarcity of representation.

The SL model trained on the SMOTE-augmented dataset outperformed the model trained on the original data. The overall accuracy reached 0.91, with significant improvements in the precision and recall for the minority classes. This performance highlights the effectiveness of combining the SMOTE for data balancing and Optuna for optimizing the ensemble of models.

### 7.3. Performance Metrics

The performance of the proposed ML model was evaluated using four metrics—accuracy, precision, recall, and F score—which are commonly used in classification problems. These metrics were computed as follows:

(5) $$\mathrm{Accuracy}=\frac{\left|TN|+|TP\right|}{\left|FN|+|FP|+|TN|+|TP\right|}$$

(6) $$\mathrm{Precision}=\frac{\left|TP\right|}{\left|FP|+|TP\right|}$$

(7) $$\mathrm{Recall}=\frac{\left|TP\right|}{\left|FN|+|TP\right|}$$

(8) $$F\;\mathrm{Score}=2\times \frac{\mathrm{Recall}\times \mathrm{Precision}}{\mathrm{Recall}+\mathrm{Precision}}$$

Here, TN, TP, FN, and FP represent true negative, true positive, false negative, and false positive, respectively. Higher values of these metrics indicate a model with accurate predictive capabilities:

- **True negative (TN)**: A TN is an outcome where the model correctly identifies non-carotid artery diseases.
- **True positive (TP)**: A TP is an outcome where the model correctly identifies carotid artery diseases.
- **False negative (FN)**: An FN is an outcome where the model incorrectly identifies non-carotid artery diseases.
- **False positive (FP)**: An FP is an outcome where the model incorrectly identifies carotid artery diseases.

Accordingly, it should be emphasized that for the above performance metrics, values closer to one indicate a model with high predictive accuracy.

## 8. Conclusions

Carotid artery diseases are associated with high mortality rates, making early diagnosis and prevention critical. The use of AI techniques, as demonstrated in this study, holds significant potential in assisting with diagnosis and reducing mortality. Despite their success in healthcare, AI systems face certain limitations, largely influenced by the quality and quantity of data available. The accuracy of these models depends on the relevance of the data used, and irrelevant or insufficient data can lead to suboptimal performance.

In this study, we explored the relationship between structural deterioration in vascular branches and carotid artery diseases such as stenosis, aneurysm, and dissection. Specifically, the diameters of common, internal, and external carotid arteries were selected as key features for data preparation in constructing the machine learning model to diagnose ischemic events. The rationale behind this was the direct influence of blood flow patterns on vessel walls, which impact endothelial cells and contribute to disease development. Therefore, the angles and branching geometry of arteries play a vital role in the creation of predictive AI models.

We utilized a combination of feature selection methods to eliminate non-informative predictors, making the developed model more interpretable and computationally efficient. The feature selection process revealed a strong connection between the structural properties of vessels and carotid artery diseases. Additionally, we developed a super learner (SL) model based on ensemble methods, including random forests (RF), AdaBoost, XGBoost, and LightGBM, to diagnose carotid artery diseases. Optuna optimization was employed to fine-tune the hyperparameters, ensuring minimal generalization error. The final SL model, combining AdaBoost, LightGBM, and RF, exhibited the best performance in diagnosing these diseases, as detailed in Table 6. The findings indicate that (1) the structural properties of carotid arteries are closely linked to disease, and (2) the developed feature selection and model architecture have strong potential for accurate disease diagnosis and prediction.

This study also presented a comprehensive analysis of machine learning techniques applied to medical datasets, emphasizing model robustness and generalization. Methods such as k-fold cross-validation, bootstrapping, data augmentation, and the SMOTE were particularly effective in improving model performance on small datasets. The siper learner ensemble model achieved 90% accuracy, with the SMOTE-augmented data significantly improving the prediction accuracy for minority classes like aneurysms and dissections. Optuna’s optimization further confirmed the advantages of data balancing and ensemble learning techniques for enhancing diagnostic capabilities.

### Future Directions

This study is structured in three stages. The first stage, presented in this manuscript, focused on creating an initial machine learning model using a dataset of 122 instances, which was expanded to 1000 instances using the SMOTE without compromising data balance. This significantly improved the model’s ability to classify minority cases.

The second stage will involve automating data extraction using deep learning techniques as more data become available. These processes will be integrated into the existing model to improve its accuracy and scalability.

The final stage will focus on integrating the machine learning model with deep learning-based data extraction, creating a comprehensive system which can process large-scale medical data and provide accurate diagnostic insights automatically. This integration will aim to refine the overall system’s efficiency and accuracy, offering valuable contributions to medical AI applications.

## Figures and Tables

**Table 1:** Demographic information about normal cases.

Sex		16 Female, 15 Male		
**Attribute**	**Minimum**	**Maximum**	**Mean**	**STD**
Age	30.000	79.000	55.452	14.156
$\cos\theta^{\prime }$–cosθ	−0.456	0.165	−0.102	0.153
cosθ (measured)	0.674	0.994	0.902	0.081
ICAdiaR (mm)	4.400	8.000	5.703	0.812
Car.Angle.Lα	12.000	58.000	27.200	12.639
Car.Angle.ΣR	20.800	76.000	46.652	14.934
Car.Angle.ΣL	26.000	105.400	50.965	19.198
Car.Angle.θR	6.300	54.100	23.052	11.427
Car.Angle.θL	4.700	49.000	23.765	12.480
Car.Angle.(R+L)θ	6.100	47.650	23.408	10.836
CCAdiaR (mm)	5.100	10.700	7.161	1.049

**Table 2:** Demographic information about stenosis cases.

Sex		10 Female, 20 Male		
**Attribute**	**Minimum**	**Maximum**	**Mean**	**STD**
Age	52.000	70.000	63.733	4.948
$\cos\theta^{\prime }$–cosθ	−0.458	0.093	−0.177	0.140
cosθ (measured)	0.592	0.987	0.919	0.079
ICAdiaR (mm)	3.500	10.500	5.707	1.484
Car.Angle.Lα	−67.300	45.000	16.767	20.079
Car.Angle.ΣR	22.600	66.400	41.510	10.079
Car.Angle.ΣL	10.700	83.900	42.243	16.088
Car.Angle.θR	3.000	36.600	17.440	7.680
Car.Angle.θL	4.000	78.000	25.477	15.115
Car.Angle.(R+L)θ	9.350	53.700	23.458	9.511
CCAdiaR (mm)	5.900	11.100	7.748	1.898

**Table 3:** Demographic information about aneurysms cases.

Sex		13 Female, 17 Male		
**Attribute**	**Minimum**	**Maximum**	**Mean**	**STD**
Age	33.000	74.000	53.167	11.680
$\cos\theta^{\prime }$–cosθ	−0.391	0.764	0.101	0.258
cosθ (measured)	0.479	0.990	0.845	0.119
ICAdiaR (mm)	3.600	9.400	6.693	1.371
Car.Angle.Lα	0.000	46.900	21.083	11.115
Car.Angle.ΣR	29.200	125.000	53.997	19.547
Car.Angle.ΣL	24.100	102.600	52.317	17.026
Car.Angle.θR	4.300	67.100	28.763	13.267
Car.Angle.θL	8.100	63.800	31.233	14.587
Car.Angle.(R+L)θ	8.050	61.400	29.998	12.894
CCAdiaR (mm)	4.900	10.800	7.393	1.353

**Table 4:** Demographic information about dissection cases.

Sex		18 Female, 13 Male		
**Attribute**	**Minimum**	**Maximum**	**Mean**	**STD**
Age	27.000	76.000	48.710	10.558
$\cos\theta^{\prime }$–cosθ	−0.429	0.256	−0.817	0.177
cosθ (measured)	0.684	0.976	0.884	0.082
ICAdiaR (mm)	3.300	6.700	4.877	0.978
Car.Angle.Lα	1.000	61.200	22.094	12.735
Car.Angle.ΣR	23.800	99.900	49.629	16.831
Car.Angle.ΣL	25.200	93.800	51.545	16.909
Car.Angle.θR	7.200	40.800	23.516	8.299
Car.Angle.θL	7.400	55.900	29.452	12.408
Car.Angle.(R+L)θ	12.700	46.850	26.484	9.458
CCAdiaR (mm)	4.900	8.900	6.261	0.888

**Table 5:** Demographic information about all cases.

Sex		57 Female, 65 Male		
**Attribute**	**Minimum**	**Maximum**	**Mean**	**STD**
Age	27.000	79.000	55.213	12.074
$\cos\theta^{\prime }$–cosθ	−0.458	0.764	−0.065	0.211
cosθ (measured)	0.479	0.994	0.888	0.095
ICAdiaR (mm)	3.300	10.500	5.738	1.340
Car.Angle.Lα	−67.300	61.200	21.833	14.831
Car.Angle.ΣR	20.800	125.000	47.950	16.185
Car.Angle.ΣL	10.700	105.400	49.300	17.618
Car.Angle.θR	3.000	67.100	23.194	11.032
Car.Angle.θL	4.000	78.000	27.467	13.841
Car.Angle.(R+L)θ	6.100	61.400	25.331	11.101
CCAdiaR (mm)	4.900	11.100	7.129	1.418

**Table 6:** Performance of the ML algorithms after optimization.

Model	Accuracy
XGBoost	0.81
LightGBM	0.86
Random Forests	0.81
Bagging	0.86
AdaBoost	0.81
ExtraTrees	0.71

**Table 7:** Classification report for the SL model.

Classes	Precision	Recall	F Score
Normal	1.0	1.0	1.0
Stenosis	0.83	0.83	0.83
Aneurysms	0.83	1.0	0.91
Dissection	1.0	0.80	0.89

**Table 8:** Demographic information for SMOTE-augmented data.

Sex		480 Female, 520 Male		
**Attribute**	**Minimum**	**Maximum**	**Mean**	**STD**
Age	27.000	79.000	52.329	15.433
$\cos\theta^{\prime }$–cosθ	−0.457	0.763	0.163	0.353
cosθ (measured)	0.479	0.993	0.735	0.146
ICAdiaR (mm)	3.302	10.499	6.883	2.085
Car.Angle.Lα	−66.166	61.186	−2.431	36.704
Car.Angle.ΣR	20.800	124.981	73.300	30.115
Car.Angle.ΣL	10.758	105.352	58.443	28.110
Car.Angle.θR	3.187	67.070	34.791	18.662
Car.Angle.θL	4.100	77.959	42.268	21.762
Car.Angle.(R+L)θ	6.249	61.368	32.789	15.736
CCAdiaR (mm)	4.901	11.093	8.051	1.769

**Table 9:** Model accuracies on the original dataset.

Model	Accuracy (Original Data)
XGBoost	0.66
LightGBM	0.76
Random Forests	0.71
Bagging	0.57
AdaBoost	0.52
ExtraTrees	0.71

**Table 10:** Model accuracies after applying SMOTE.

Model	Accuracy (SMOTE Data)
XGBoost	0.81
LightGBM	0.86
Random Forests	0.81
Bagging	0.86
AdaBoost	0.81
ExtraTrees	0.71

**Table 11:** Comparison of model accuracies before and after SMOTE.

Model	Accuracy (Original)	Accuracy (SMOTE)
XGBoost	0.66	0.81
LightGBM	0.76	0.86
Random Forests	0.71	0.81
Bagging	0.57	0.86
AdaBoost	0.52	0.81
ExtraTrees	0.71	0.71

**Table 12:** Accuracy of models on SMOTE-expanded dataset after Optuna optimization.

Model	Accuracy (SMOTE Data + Optuna)
XGBoost	0.89
LightGBM	0.91
Random Forests	0.88
Bagging	0.90
AdaBoost	0.87
ExtraTrees	0.79

**Table 13:** Comparison of model accuracies: original Ddata + Optuna vs. SMOTE data + Optuna.

Model	Accuracy (Original Data + Optuna)	Accuracy (SMOTE Data + Optuna)
XGBoost	0.81	0.89
LightGBM	0.86	0.91
Random Forests	0.81	0.88
Bagging	0.86	0.90
AdaBoost	0.81	0.87
ExtraTrees	0.71	0.79

**Table 14:** Classification report for the SL model on SMOTE-augmented data.

Classes	Precision	Recall	F Score
Normal	1.0	1.0	1.0
Stenosis	0.88	0.86	0.87
Aneurysm	0.90	1.0	0.95
Dissection	1.0	0.85	0.92

## Data Availability

Data are contained within the article.

## References

[1] Campbell B.C.V., Silva D.A.D., Macleod M.R., Coutts S.B., Schwamm L.H., Davis S.M., Donnan G.A. (2019). Ischaemic stroke. Nat. Rev. Dis. Prim..

[2] Shiber J.R., Fontane E., Adewale A. (2010). Stroke registry: Hemorrhagic vs ischemic strokes. Am. J. Emerg. Med..

[3] Sacco R.L. (1997). Risk factors, outcomes, and stroke subtypes for ischemic stroke. Neurology.

[4] Romero J.R. (2007). Prevention of Ischemic Stroke: Overview of Traditional Risk Factors. Curr. Drug Targets.

[5] Pandian J.D., Gall S.L., Kate M.P., Silva G.S., Akinyemi R.O., Ovbiagele B.I., Lavados P.M., Gandhi D.B.C., Thrift A.G. (2018). Prevention of stroke: A global perspective. Lancet.

[6] Rittenhouse E.A., Radke H.M., Sumner D.S. (1972). Carotid Artery Aneurysm: Review of the Literature and Report of a Case With Rupture Into the Oropharynx. Arch. Surg..

[7] Bahram M. (1997). Spontaneous dissections of internal carotid arteries. Neurologist.

[8] Murray C.D. (1926). The physiological principle of minimum work applied to the angle of branching of arteries. J. Gen. Physiol..

[9] Murray C.D. (1927). A relationship between circumference and weight in trees and its bearing on branching angles. J. Gen. Physiol..

[10] Prasad K.M., Radhakrishnamacharya G. (2008). Flow of Herschel-Bulkley fluid through an inclined tube of nonuniform cross-section with multiple stenosis. Arch. Mech..

[11] Dhange M., Sankad G., Safdar R., Jamshed W., Eid M.R., Bhujakkanavar U., Gouadria S., Chouikh R. (2022). A mathematical model of blood flow in a stenosed artery with post-stenotic dilatation and a forced field. PLoS ONE.

[12] Sun J., Guo L., Jing J., Tang C., Lu Y., Fu J., Ullmann A., Brauner N. (2021). Investigation on laminar pipe flow of a non-Newtonian Carreau-Extended fluid. J. Pet. Sci. Eng..

[13] Reid L., Rea P.M. (2021). An Introduction to Biomedical Computational Fluid Dynamics. Biomedical Visualisation: Volume 10.

[14] Apaydin M., Cetinoglu K. (2021). Carotid angle in young stroke. Clin. Imaging.

[15] Noh S.M., Kang H.G. (2019). Clinical significance of the internal carotid artery angle in ischemic stroke. Sci. Rep..

[16] Ojaare1 M.G., Annougu T.I., Msuega C.D., Mohammad H.O., Farati A., Alexander A., Umer B.P. (2021). Carotid artery diameter assessment in men and women and the relation to age, sex and body mass index using ultrasonography. Int. J. Adv. Med..

[17] Tan Q., Qin C., Yang J., Wang T., Lin H., Lin C., Chen X. (2021). Inner diameters of the normal carotid arteries measured using three-dimensional digital subtraction catheter angiography: A retrospective analysis. BMC Neurol..

[18] İbrahim Özdemir H. (2020). The structural properties of carotid arteries in carotid artery diseases a retrospective computed tomography angiography study. Pol. J. Radiol..

[19] Yoshida K., Yang T., Yamamoto Y., Kurosaki Y., Funaki T., Kikuchi T., Ishii A., Kataoka H., Miyamoto S. (2019). Expansive carotid artery remodeling: Possible marker of vulnerable plaque. J. Neurosurg..

[20] Le E.P., Wong M.Y., Rundo L., Tarkin J.M., Evans N.R., Weir-McCall J.R., Chowdhury M.M., Coughlin P.A., Pavey H., Zaccagna F. (2024). Using machine learning to predict carotid artery symptoms from CT angiography: A radiomics and deep learning approach. Eur. J. Radiol. Open.

[21] Porcu M., Cau R., Suri J.S., Saba L. (2022). Artificial intelligence-and radiomics-based evaluation of carotid artery disease. Artificial Intelligence in Cardiothoracic Imaging.

[22] Saba L., Chen H., Cau R., Rubeis G., Zhu G., Pisu F., Jang B., Lanzino G., Suri J., Qi Y. (2022). Impact analysis of different CT configurations of carotid artery plaque calcifications on cerebrovascular events. Am. J. Neuroradiol..

[23] Goodfellow I.J., Bengio Y., Courville A. (2016). Deep Learning.

[24] Pisu F., Chen H., Jiang B., Zhu G., Usai M.V., Austermann M., Shehada Y., Johansson E., Suri J., Lanzino G. (2024). Machine learning detects symptomatic patients with carotid plaques based on 6-type calcium configuration classification on CT angiography. Eur. Radiol..

[25] Cohen I., Huang Y., Chen J., Benesty J., Benesty J., Chen J., Huang Y., Cohen I. (2009). Pearson correlation coefficient. Noise Reduction in Speech Processing.

[26] Tallarida R.J., Murray R.B., Tallarida R.J., Murray R.B. (1987). Chi-square test. Manual of Pharmacologic Calculations: With Computer Programs.

[27] Colan S.D. (2013). The why and how of Z scores. J. Am. Soc. Echocardiogr..

[28] Fonti V., Belitser E. (2017). Feature selection using lasso. VU Amsterdam Res. Pap. Bus. Anal..

[29] Chen X.w., Jeong J.C. Enhanced recursive feature elimination. Proceedings of the Sixth International Conference on Machine Learning and Applications (ICMLA 2007).

[30] İbrahim Özdemir H., Çınar C., İsmail O. (2021). Determination of hemodynamic and rheological properties in carotid artery diseases. Imaging Med..

[31] John G.H., Kohavi R., Pfleger K., Cohen W.W., Hirsh H. (1994). Irrelevant Features and the Subset Selection Problem. Machine Learning Proceedings 1994.

[32] Saeys Y., Inza I., Larrañaga P. (2007). A review of feature selection techniques in bioinformatics. Bioinformatics.

[33] Harrell F. (2015). Regression Modeling Strategies: With Applications to Linear Models, Logistic and Ordinal Regression, and Survival Analysis.

[34] Akiba T., Sano S., Yanase T., Ohta T., Koyama M. Optuna: A Next-generation Hyperparameter Optimization Framework. Proceedings of the 25th ACM SIGKDD International Conference on Knowledge Discovery & Data Mining.

[35] Chen T., Guestrin C. XGBoost: A Scalable Tree Boosting System. Proceedings of the 22nd ACM SIGKDD International Conference on Knowledge Discovery and Data Mining.

[36] Ke G., Meng Q., Finley T., Wang T., Chen W., Ma W., Ye Q., Liu T.Y. (2017). Lightgbm: A highly efficient gradient boosting decision tree. Adv. Neural Inf. Process. Syst..

[37] Ho T.K. Random decision forests. Proceedings of the 3rd International Conference on Document Analysis and Recognition.

[38] Breiman L. (2004). Bagging predictors. Mach. Learn..

[39] Breiman L. (1999). Pasting Small Votes for Classification in Large Databases and On-Line. Mach. Learn..

[40] Louppe G., Geurts P. Ensembles on Random Patches. Proceedings of the Machine Learning and Knowledge Discovery in Databases.

[41] Laan M., Polley E., Hubbard A. (2007). Super Learner. Stat. Appl. Genet. Mol. Biol..

[42] Nasarian E., Abdar M., Fahami M.A., Alizadehsani R., Hussain S., Basiri M.E., Zomorodi-Moghadam M., Zhou X., Pławiak P., Acharya U.R. (2020). Association between work-related features and coronary artery disease: A heterogeneous hybrid feature selection integrated with balancing approach. Pattern Recognit. Lett..

